# Antibiofilm, AntiAdhesive and Anti-Invasive Activities of Bacterial Lysates Extracted from *Pediococcus acidilactici* against *Listeria monocytogenes*

**DOI:** 10.3390/foods11192948

**Published:** 2022-09-21

**Authors:** Han Bin Lee, Ki Hwan Kim, Gweon Ah Kang, Kwang-Geun Lee, Seok-Seong Kang

**Affiliations:** Department of Food Science and Biotechnology, College of Life Science and Biotechnology, Dongguk University-Seoul, Goyang 10326, Korea

**Keywords:** *Pediococcus acidilactici*, bacterial lysates, *Listeria monocytogenes*, biofilm, adhesion, invasion

## Abstract

This study aimed to investigate whether bacterial lysates (BLs) extracted from *Pediococcus acidilactici* reduce *Listeria monocytogenes* biofilm formation, as well as adhesion to and invasion of human intestinal epithelial cells. Pretreatment with *P. acidilactici* BLs (20, 40, and 80 μg/mL) significantly inhibited *L. monocytogenes* biofilm formation on the surface of polystyrene (*p* < 0.05). Fluorescence and scanning-electron-microscopic analyses indicated that *L. monocytogenes* biofilm comprised a much less dense layer of more-dispersed cells in the presence of *P. acidilactici* BLs. Moreover, biofilm-associated genes, such as *flaA*, *fliG*, *flgE*, *motB*, *degU*, *agrA*, and *prfA*, were significantly downregulated in the presence of *P. acidilactici* BLs (*p* < 0.05), suggesting that *P. acidilactici* BLs prevent *L. monocytogenes* biofilm development by suppressing biofilm-associated genes. Although *P. acidilactici* BLs did not dose-dependently inhibit *L. monocytogenes* adhesion to and invasion of intestinal epithelial cells, the BLs effectively inhibited adhesion and invasion at 40 and 80 μg/mL (*p* < 0.05). Supporting these findings, *P. acidilactici* BLs significantly downregulated *L. monocytogenes* transcription of genes related to adhesion and invasion, specifically *fbpA*, *ctaP*, *actA*, *lapB*, *ami*, and *inlA*. Collectively, these results suggest that *P. acidilactici* BLs have the potential to reduce health risks from *L. monocytogenes*.

## 1. Introduction

*Listeria monocytogenes* is frequently found in soil, vegetation, and animals and causes serious diseases, including listeriosis [1]. Although listeriosis is less common than other foodborne diseases, more than 30% of mortality has been reported in vulnerable groups, such as pregnant women, newborns, and the elderly [2]. *L. monocytogenes* can survive in acidic or salty conditions and multiply slowly at the low temperatures typical of food-processing environments. Consequently, listeriosis is often caused by ingestion of contaminated food sources, such as dairy products, vegetables, meat products, and ready-to-eat foods [3]. 

Many foodborne pathogenic bacteria, including *L. monocytogenes*, are known to form biofilms on food-contact surfaces such as polystyrene, glass, and stainless steel, as well as on biotic surfaces [4]. Biofilms consist of a structured community of bacterial cells embedded in the matrix of extracellular polymeric substances produced by bacteria [5]. The molecular mechanisms underlying the regulation of biofilm formation of *L. monocytogenes* have been widely studied. For instance, the ability of *L. monocytogenes* to adhere to surfaces in the early period of biofilm formation is facilitated by flagellin [6]. Genes involved in flagellar synthesis and motility that enable the adhesion of *L. monocytogenes* to surfaces include *flaA*, *fliG*, *flgE*, *motB*, and *degU* [7,8]. In biofilm formation of *L. monocytogenes*, genes related to quorum sensing and virulence, such as *agrA* and *prfA*, are also involved [9,10].

Compared to their planktonic counterparts, bacterial cells in a biofilm are highly resistant to chemical and physical stresses, such as heat, desiccation, acidic conditions, and antibiotics [11]. Although biofilm formation of *L. monocytogenes* on food-contact surfaces is considered a critical pathway for pathogenic resistance, inhibiting or eradicating *L. monocytogenes* biofilm is difficult during food processing. For instance, *L. monocytogenes* biofilm that was repeatedly exposed to sanitizers developed resistance to those sanitizing agent [12]. 

Once ingested, *L. monocytogenes* binds to receptors on the surface of intestinal epithelial cells and disseminates to various tissues via the bloodstream [13]. Adhesion of *L. monocytogenes* to intestinal epithelial cells is required for initiating infection and promoting bacterial dissemination to extraintestinal sites [14]. Bacterial adhesion is also a prerequisite for biofilm development [15]. It has been known that several factors of *L. monocytogenes*, including fibronectin binding protein, are associated with the bacterial adhesion to intestinal epithelial cells. In addition, other proteins, including CtaP, ActA, LapB, and Ami, are also attributed to the adhesion of *L. monocytogenes* to the intestinal epithelial cells [16]. 

Numerous efforts have been carried out to find natural antimicrobials that can act as alternatives to chemical additives in preserving food and preventing infection. Among the natural antimicrobials, probiotic lactic acid bacteria have attracted strong interest for their ability to inhibit foodborne pathogenic bacteria. Since lactic acid bacteria are considered “generally recognized as safe” and produce various antimicrobial substances, their antimicrobial activity has been widely examined [17]. However, live probiotic microorganisms have certain limitations, such as antibiotic resistance and the potential for infection in immunocompromised individuals [18]. Currently, postbiotics, such as metabolites from cell-free supernatants, exopolysaccharides, cell-wall fragments, and bacterial lysates (BLs), have emerged as a promising alternative to probiotics. *Pediococcus acidilactici* is recognized as a potential probiotic bacterium with antimicrobial activities [19]. Many probiotic lactobacilli and their postbiotic compounds have shown antagonistic activities against the growth and biofilm formation of *L. monocytogenes* [20,21,22]. In addition, it has been widely documented that bacteriocins produced by *P. acidilactici* suppress the growth of *L. monocytogenes* [23,24,25]. However, the antibiofilm, antiadhesion, and anti-invasion activities of *P. acidilactici* BLs against *L. monocytogenes* have not been elucidated. Therefore, in this study, we investigated the inhibitory effects of BLs extracted from two *P. acidilactici* strains, K10 and HW01, on *L. monocytogenes* mediated biofilm formation on an abiotic surface, as well as *L. monocytogenes* adhesion to and invasion of human intestinal epithelial cells. 

## 2. Materials and Methods

### 2.1. Bacterial Culture and BL Preparation

Two strains of *P. acidilactici* (K10 and HW01) were cultured in Man−Rogosa−Sharpe broth (Neogen, Lansing, MI, USA) at 37 °C. To prepare *P. acidilactici* BLs, bacterial pellets were obtained from overnight cultures of *P. acidilactici* K10 and *P. acidilactici* HW01 that were centrifugated at 8000 × *g* for 10 min and vigorously washed with phosphate-buffered saline (PBS). The bacterial pellets were then resuspended in an extraction buffer (50 mM Trizma base, 0.1 mM EDTA, and 1 mM 2-mercaptoethanol, pH 7.5) and homogenized in a tube containing zirconium beads (Benchmark Scientific, Sayreville, NJ, USA) at 4 °C for 100 s using a benchtop homogenizer (BeadBug^TM^ 3, Benchmark Scientific, Sayreville, NJ, USA). After centrifugation at 20,000 × *g* for 30 min, *P. acidilactici* BLs were obtained and their concentration was measured using a bicinchoninic acid protein assay (Thermo Scientific, Rockford, IL, USA). *L. monocytogenes* KCTC 3569 was purchased from the Korean Collection for Type Cultures (Jeongeup, Korea) and cultured in Brain Heart Infusion (BHI) broth (BD Biosciences, Franklin Lakes, NJ, USA) at 37 °C. For subsequent experiments, *L. monocytogenes* cultures were diluted to 1 × 10^7^ colony-forming units (CFU) per mL, corresponding to 0.1 at 600 nm of optical density (OD) in BHI broth.

### 2.2. Inhibition of L. monocytogenes Biofilm Formation 

BLs extracted from *P. acidilactici* K10 (K10 BL) or *P. acidilactici* HW01 (HW01 BL) (20, 40, or 80 μg/mL) were added to wells of a 96-well culture plate and pre-incubated for 3 h at 37 °C. *L. monocytogenes* (1 × 10^7^ CFU/mL) was then added to wells of the culture plate and incubated for an additional 24 h. After incubation, planktonic *L. monocytogenes* was removed, and *L. monocytogenes* biofilm was gently washed with PBS. The biofilm was stained with 0.1% crystal violet for 30 min at room temperature and washed with PBS twice to remove excess stain. The adherent stain was dissolved with 0.1% acetic acid and 95% ethanol, after which the biofilm was quantified by measuring the OD at 595 nm using a microplate reader (AMR-100, Allsheng, Hangzhou, China). 

### 2.3. Microscopic Analysis

For confocal laser scanning microscopy (CLSM), K10 BL or HW01 BL (20, 40, or 80 μg/mL) was added to cover glass-bottom dishes (SPL Life Science, Pocheon, Korea) and preincubated at 37 °C for 3 h. *L. monocytogenes* (1 × 10^7^ CFU/mL) was then added and incubated at 37 °C for an additional 24 h. Planktonic *L. monocytogenes* was removed by washing with PBS, and *L. monocytogenes* biofilm was stained using a LIVE/DEAD BacLight bacterial viability kit (Molecular Probes, Eugene, OR, USA) according to the manufacturer’s instructions. The biofilm was visualized with a confocal laser scanning microscope (Eclipse Ti-E, Nikon, Tokyo, Japan). To analyze the *L. monocytogenes* biofilm with a scanning electron microscope (SEM), K10 BL or HW01 BL (20, 40, or 80 μg/mL) was added to a 12 mm coverslip (SPL Life Science) in a 24-well culture plate and preincubated at 37 °C for 3 h, followed by incubation with *L. monocytogenes* (1 × 10^7^ CFU/mL) at 37 °C for an additional 24 h. After incubation, the *L. monocytogenes* biofilm was gently washed with PBS and fixed with 2.5% glutaraldehyde and 2% paraformaldehyde in PBS at 4 °C overnight. The biofilm was then rinsed with PBS and dehydrated by washing with different concentrations of ethanol (70, 80, 90, 95, and 99%, each for 15 min). After drying with hexamethyldisilazane for 30 min, the coverslip was coated with ion sputter. The *L. monocytogenes* biofilm was examined using a scanning electron microscope (Carl Zeiss, Oberkochen, Germany). 

### 2.4. Viability of Planktonic L. monocytogenes 

To measure the viability of planktonic *L. monocytogenes*, *L. monocytogenes* was cultured in the presence or absence of K10 BL or HW01 BL (20, 40, or 80 μg/mL) in a tube at 37 °C for 24 h. After the serial dilution, the viability of planktonic *L. monocytogenes* was determined by plating samples on BHI agar. 

### 2.5. Adhesion and Invasion Assay

To investigate the inhibitory effects of *P. acidilactici* BLs on the adhesion and invasion of *L. monocytogenes* to HT-29 cells, a human-intestinal-epithelial cell line, HT-29 cells were cultured in Dulbecco’s modified Eagle’s medium (DMEM; Welgene, Gyeongsan, Korea) supplemented with 10% fetal-bovine serum, 100 U/mL penicillin, and 100 μg/mL streptomycin (HyClone, Logan, UT, USA) at 37 °C in a 5% CO_2_ humidified incubator. To conduct the adhesion assay, HT-29 cells (5 × 10^5^ cells/mL) were plated in a 12-well culture plate and incubated until the cells were fully confluent. After washing the cell monolayers with PBS, the cells were pretreated with K10 BL or HW01 BL (20, 40, or 80 μg/mL) at 37 °C for 3 h and incubated with *L. monocytogenes* (1 × 10^7^ CFU/mL) for 1 h in antibiotic-free DMEM. The control cells were incubated with *L. monocytogenes* without the pretreatment with K10 BL or HW01 BL. HT-29 cells were gently washed with PBS to remove nonadherent *L. monocytogenes* and lysed with 0.2% Triton X-100 at 4 °C for 20 min. The lysed HT-29 cells were serially diluted, and the adherent *L. monocytogenes* was enumerated by plating samples on BHI agar. For the invasion assay, HT-29 cells were cultured in DMEM supplemented with 10% fetal-bovine serum, 100 U/mL penicillin, and 100 μg/mL streptomycin (HyClone), as described above. The cell monolayers were gently washed with PBS. The cells were pretreated with K10 BL or HW01 BL (20, 40, or 80 μg/mL) at 37 °C for 3 h and incubated with *L. monocytogenes* (1 × 10^7^ CFU/mL) for 1 h in antibiotic-free DMEM. After incubation with *L. monocytogenes*, HT-29 cells were gently washed with PBS and incubated in DMEM supplemented with gentamicin (100 μg/mL) at 37 °C for 1 h to kill the remaining extracellular *L. monocytogenes*. Then, HT-29 cells were lysed with 0.2% Triton X-100, and the extent of the *L. monocytogenes* invasion was determined by plating samples on BHI agar. 

### 2.6. Quantitative Reverse Transcription Polymerase Chain Reaction (qRT-PCR)

To examine the inhibitory effect of *P. acidilactici* BLs on *L. monocytogenes* biofilm, the expression of genes responsible for biofilm formation was determined by qRT-PCR. After K10 BL or HW01 BL (80 μg/mL) was incubated in a 6-well culture plate for 3 h, *L. monocytogenes* (1 × 10^7^ CFU/mL) was added to each well and incubated at 37 ℃ for an additional 24 h to allow for biofilm formation. The *L. monocytogenes* biofilm was washed with PBS and incubated with 1.5 mg/mL lysozyme (Sigma-Aldrich, St. Louis, MO, USA) at 37 °C for 30 min. Total RNA was extracted from *L. monocytogenes* biofilm cells using TRIzol^®^ Max™ Bacterial RNA Isolation Kit (Invitrogen, Carlsbad, CA, USA) according to the manufacturer’s instructions. Then, the total RNA was reverse-transcribed to complementary DNA (cDNA) using random hexamers and reverse transcriptase (Promega, Madison, WI, USA). The cDNA was amplified by qRT-PCR with SYBR Green Real-Time PCR master mix (Toyobo, Osaka, Japan) using the StepOnePlus^TM^ real-time PCR system (Applied Biosystems, Foster City, CA, USA). The cycling conditions of qRT-PCR consisted of an initial denaturation step at 95 °C for 10 s, followed by amplification for 40 cycles at 95 °C for 5 s and at 60 °C for 31 s. The primer sequences used for genes responsible for biofilm formation are as follows: *flaA*, forward, 5′-CTGGTATGAGTCGCCTTAG-3′ and reverse, 5′-CATTTGCGGTGTTTGGTTTG-3′; *fliG*, forward, 5′-CCGCCCTTATTATTTGGAGC-3′ and reverse, 5′-CGAGTTTAGCAATTCCTCCTG-3′; *flgE*, forward, 5′-AATGCCAACACGACAGGATA-3′ and reverse, 5′-TTTGTTCCAGCGTAAAGTCC-3′; *motB*, forward, 5′-TGCAAAAAAATTCGAACAAATGG-3′ and reverse, 5′-CTGCCGCGCCTTCCT-3′; *degU*, forward, 5′-ACGCATAGAGAGTGCGA GGTATT-3′ and reverse, 5′-CCCAATTCCGCGGTTACTT-3′; *agrA*, forward, 5′-ATGAAGCAAGCGGAAGAAC-3′ and reverse, 5′-TACGACCTGTGACAACGATAAA-3′; and *prfA*, forward, 5′-ATGAACGCTCAAGCAGAAGA-3′ and reverse, 5′-CGAAAGCACCTTTGTAGTATTG-3′. In a separate experiment, genes related to adhesion and invasion in *L. monocytogenes*, specifically *fbpA*, *ctaP*, *actA*, *lapB*, *ami*, and *inlA*, were amplified as described above. The primer sequences used are as follows: *fbpA*, forward, 5′- AAGCGACTTTACCTGCTCCA-3′ and reverse, 5′- AACCAGGCAAATCTTTCACG-3′; *ctaP*, forward, 5′- GCAGACTACTCTATCGCACTAAATGG-3′ and reverse, 5′- GATTTCTTGACGTTCTTTGTCGTCAGC-3′; *actA*, forward, 5′- CGACCGACCARCTMTRCAAGTG-3′ and reverse, 5′- TCCGCKGCGCTATCC-3′; *lapB*, forward, 5′- TGGAGTGGGCACGTGTTGT-3′ and reverse, 5′- TTGTCAGCTGCATATTGTGAATTG-3′; *ami*, forward, 5′- TGGGGAGCAGGACAATATGC-3′ and reverse, 5′- CAGTATGGGTTGTTCCGCCT-3′; and *inlA*, forward, 5′-ACTTGGCAGTGGAGTATGGA-3′ and reverse, 5′-CTGAAGCGTCGTAACTTGGTC-3′. An internal standard for all qRT-PCR, 16s rRNA, was amplified using the following primer sequence: forward, 5′-ACCGTCAAGGGACAAGCA-3′ and reverse, 5′-GGGAGGCAGCAGTAGGGA-3′. The 2^−ΔΔCt^ method was used to normalize relative gene expression to 16 s rRNA.

### 2.7. Statistical Analysis

All data presented in this study were obtained from three independent experiments. Statistical significance was determined in comparison to the appropriate control by performing a one-way analysis of variance (ANOVA) using IBM SPSS Statistics 25 software (IBM, Armonk, NY, USA) or by performing an unpaired two-tailed *t*-test using GraphPad Prism 5 (GraphPad Software Inc., La Jolla, CA, USA) when *p* < 0.05. 

## 3. Results 

### 3.1. P. acidilactici BLs Inhibit L. monocytogenes Biofilm Formation

**Figure 1:**
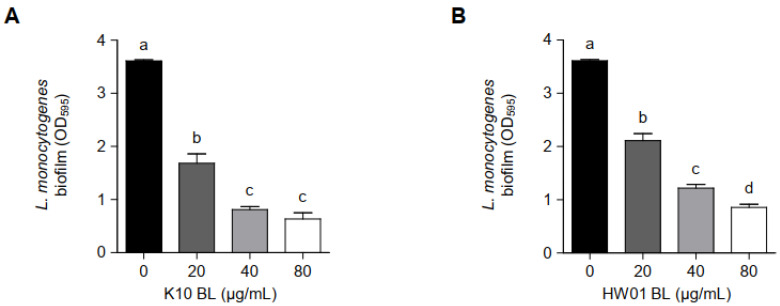
Inhibitory effect of *P. acidilactici* BLs on *L. monocytogenes* biofilm formation. K10 BL (**A**) or HW01 BL (**B**) (0, 20, 40, and 80 μg/mL) was preincubated at 37 °C for 3 h in a 96-well culture plate, after which *L. monocytogenes* (1 × 10^7^ CFU/mL) was added and incubated at 37 °C for an additional 24 h. *L. monocytogenes* biofilm formation was assessed by crystal violet staining. The results are represented as the means ± standard deviations from three independent experiments (*p* < 0.05). Significant differences between treatment with K10 BL or HW01 BL are expressed with different letters (a–d).

**Figure 2:**
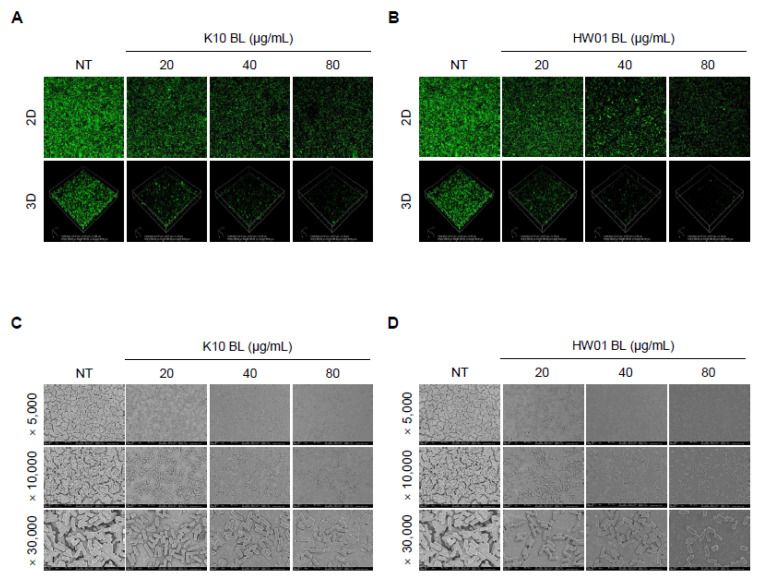
Disruption of *L. monocytogenes* biofilm formation by *P. acidilactici* BLs. K10 BL or HW01 BL (0, 20, 40, and 80 μg/mL) was preincubated at 37 °C for 3 h on cover glasses, after which *L. monocytogenes* (1 × 10^7^ CFU/mL) was added and incubated at 37 °C for an additional 24 h. Biofilm formation was then analyzed using confocal laser scanning microscopy (**A**,**B**). For scanning-electron-microscopic analysis, K10 BL or HW01 BL (0, 20, 40, and 80 μg/mL) was preincubated at 37 °C for 3 h on coverslips, and *L. monocytogenes* (1 × 10^7^ CFU/mL) was then added and incubated at 37 °C for an additional 24 h. Samples were then analyzed using scanning electron microscopy at 5000, 10,000, and 30,000× magnifications (**C**,**D**). All images taken from one of three similar results are shown.

As shown in Figure 1, pretreatment with either K10 BL or HW01 BL inhibited *L. monocytogenes* biofilm formation. K10 BL significantly inhibited *L. monocytogenes* biofilm formation compared to the untreated control (*p* < 0.05). A reduction of 53, 78, and 82% relative to the untreated control was determined at 20, 40, and 80 μg/mL of K10 BL, respectively (Figure 1A). Similarly, HW01 BL significantly reduced *L. monocytogenes* biofilm development (*p* < 0.05). HW01 BL at a concentration of 20 μg/mL was able to reduce *L. monocytogenes* biofilm formation to 42% of untreated *L. monocytogenes* biofilm formation. Furthermore, 40 and 80 μg/mL of HW01 BL markedly inhibited *L. monocytogenes* biofilm formation by 66 and 76%, respectively (Figure 1B). To further characterize the effect of *P. acidilactici* BLs on *L. monocytogenes* biofilm formation, glass coverslips were pretreated with 20, 40, or 80 μg/mL of K10 BL or HW01 BL, and *L. monocytogenes* was allowed to form a biofilm on the surface of glass coverslips. CLSM analysis revealed a dense, thick, and fully established biofilm in untreated *L. monocytogenes* samples, while *L. monocytogenes* biofilm formed in the presence of K10 BL was much less dense. At the highest K10 BL concentration (80 μg/mL), negligible biofilm mass was detected (Figure 2A). Similar to the K10 BL pretreatment, the HW01 BL pretreatment caused *L. monocytogenes* biofilm to become thinner and more dispersed (Figure 2B). Further, SEM images showed that K10 BL and HW01 BL inhibited *L. monocytogenes* biofilm formation at concentrations of 20, 40, and 80 μg/mL (Figure 2C and Figure 2D, respectively). Upon pretreatment with K10 BL or HW01 BL, *L. monocytogenes* biofilm appeared as a monolayer of dispersed cells scattered on the surface. These results demonstrate that *P. acidilactici* BLs have a remarkable effect on the inhibition of *L. monocytogenes* biofilm formation.

### 3.2. Antibiofilm Activity of P. acidilactici BL Is Not Affected by the Viability of L. monocytogenes

**Figure 3:**
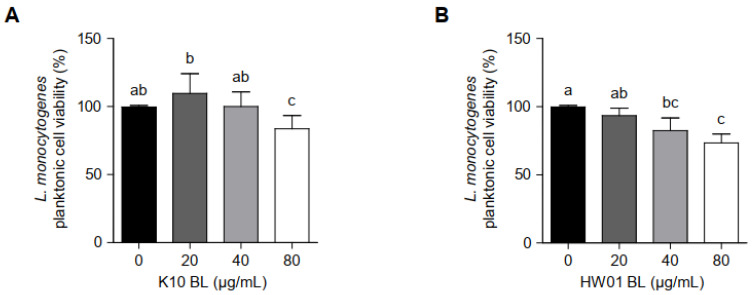
The viability of planktonic *L. monocytogenes* in the presence or absence of *P. acidilactici* BLs. Planktonic *L. monocytogenes* was incubated with K10 BL (**A**) or HW01 BL (**B**) (0, 20, 40, and 80 μg/mL) at 37 °C for 24 h. After incubation, *L. monocytogenes* was enumerated by plating samples on BHI agar. The results are represented as the means ± standard deviations from three independent experiments (*p* < 0.05). Significant differences between treatment with K10 BL or HW01 BL are expressed with different letters (a–d).

In order to examine whether *P. acidilactici* BLs inhibit *L. monocytogenes* biofilm formation by decreasing bacterial viability at planktonic states, planktonic *L. monocytogenes* cultured with different concentrations of *P. acidilactici* BLs were enumerated. As shown in Figure 3A, the viability rates of planktonic *L. monocytogenes* cultured with 20 and 40 μg/mL of K10 BL showed no significant difference compared to the control without K10 BL. However, a higher concentration of K10 BL (80 μg/mL) decreased the viability of planktonic *L. monocytogenes* by 83%. HW01 BL gradually decreased the viability of planktonic *L. monocytogenes*; however, the viability rates, which were 93, 82, and 73% at the concentrations of 20, 40, and 80 μg/mL of HW01 BL, respectively, did not decrease dose-dependently (Figure 3B). These results suggest that *P. acidilactici* BLs did not inhibit *L. monocytogenes* biofilm formation by killing bacteria in the planktonic state. 

### 3.3. P. acidilactici BLs Downregulate the Expression of Genes Critical for L. monocytogenes Biofilm Formation

**Figure 4:**
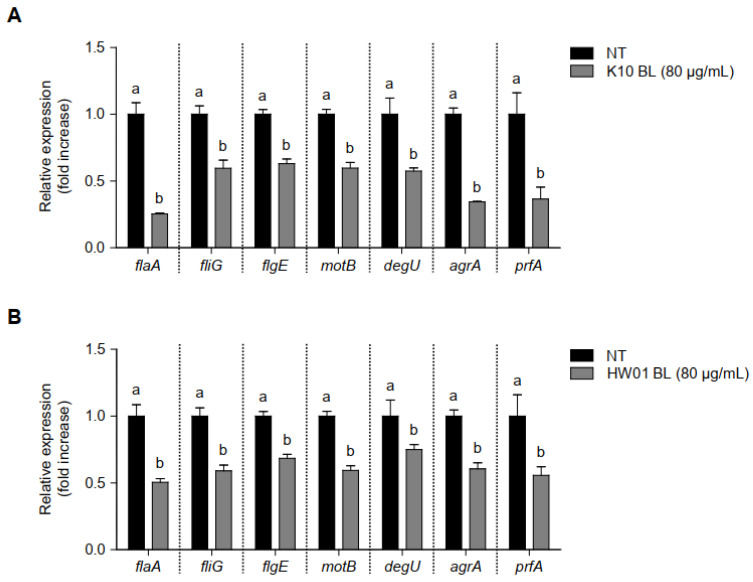
Regulation of gene transcription associated with *L. monocytogenes* biofilm formation in the presence or absence of *P. acidilactici* BLs. K10 BL (**A**) or HW01 BL (**B**) (80 μg/mL) was preincubated for 3 h in a culture plate, after which *L. monocytogenes* was added and cultured at 37 °C for an additional 24 h. The expression levels of *flaA*, *fliG*, *flgE*, *motB*, *degU*, *agrA*, and *prfA* were measured using qRT-PCR. The results are represented as the means ± standard deviations from three independent experiments (*p* < 0.05). Significant differences between treatment with K10 BL or HW01 BL are expressed with different letters (a and b).

Since several genes are related to biofilm formation and virulence of *L. monocytogenes*, mRNA expression in response to treatment with BLs was determined. As compared to the untreated control, the expressions of all genes considered in the current study were significantly downregulated in the presence of K10 BL and HW01 BL (80 μg/mL) (Figure 4A and Figure 4B, respectively). Genes involved in flagellar synthesis and motility that enable *L. monocytogenes* attachment, such as *flaA*, *fliG*, *flgE*, *motB*, and *degU*, were significantly downregulated by K10 BL and HW01 BL (*p* < 0.05). Particularly, expression of *flaA* decreased by more than 50% in the presence of both *P. acidilactici* BLs. Thus, it is likely that *P. acidilactici* BLs inhibited *L. monocytogenes* biofilm formation by interfering with bacterial attachment to the surface. The expression of *agrA* is associated with the *L. monocytogenes* quorum-sensing system, which plays a critical role in biofilm formation [26]. As shown in Figure 4A,B, both K10 BL and HW01 BL also significantly downregulated the expression of *agrA* (*p* < 0.05). The reduction of *agrA* transcription by K10 BL exceeded 60%, whereas HW01 BL inhibited *agrA* gene expression by 40%. Both K10 BL and HW01 BL significantly reduced expression of *prfA*, which is closely associated with *L. monocytogenes* biofilm formation, compared to the untreated control (*p* < 0.05). These results suggest that *P. acidilactici* BLs regulate the expression of genes that play a critical role in biofilm initiation and development in *L. monocytogenes*.

### 3.4. P. acidilactici BLs Reduce L. monocytogenes Adhesion to and Invasion of HT-29 Cells

**Figure 5:**
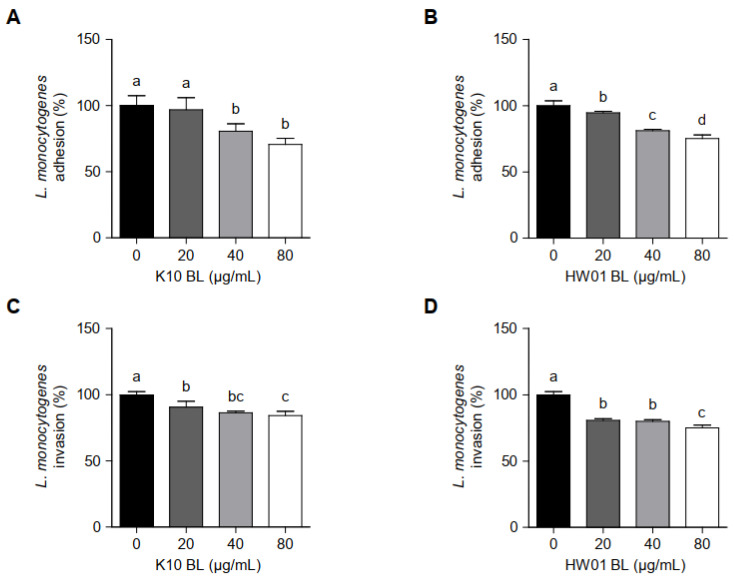
*L. monocytogenes* adhesion to and invasion of HT-29 cells in the presence or absence of *P. acidilactici* BLs. HT-29 cells were pretreated with K10 BL (**A**,**C**) or HW01 BL (**B**,**D**) (0, 20, 40, and 80 μg/mL) for 3 h, after which *L. monocytogenes* (1 × 10^7^ CFU/mL) was added and incubated for 1 h. Adhesive and invasive abilities of *L. monocytogenes* were determined by plating samples on BHI agar. The results are represented as the means ± standard deviations from three independent experiments (*p* < 0.05). Significant differences between treatment with K10 BL or HW01 BL are expressed with different letters (a–d).

*L. monocytogenes* adhesion to and invasion of intestinal epithelial cells are responsible for listeriosis. Thus, we examined whether *P. acidilactici* BLs interfere *L. monocytogenes* adhesion to and invasion of HT-29 cells. As shown in Figure 5A, adhesion of *L. monocytogenes* to HT-29 cells was not significantly reduced by K10 BL at 20 μg/mL (*p* > 0.05), although the same concentration of HW01 BL (20 μg/mL) significantly inhibited the adhesion of *L. monocytogenes* to HT-29 cells (*p* < 0.05) (Figure 5B). Further, treatment with higher concentrations (40 μg/mL) of K10 BL and HW01 BL significantly reduced *L. monocytogenes* adhesion by 19%. *L. monocytogenes* adhesion was further inhibited by 29% and 25% when the cells were treated with 80 μg/mL of K10 BL and HW01 BL, respectively (Figure 5A,B). Figure 5C displays that the invasion rates of *L. monocytogenes* were reduced to 8, 14, and 16% of those of the control, at 20, 40, and 80 μg/mL of K10 BL, respectively. Similarly, HW01 pretreatment at 20, 40, and 80 μg/mL reduced the invasion of *L. monocytogenes* at rates of 19, 20, and 25% of those of the control, respectively (Figure 5D). 

### 3.5. P. acidilactici BLs Downregulate Genes Related to the Adhesion and Invasion in L. monocytogenes

**Figure 6:**
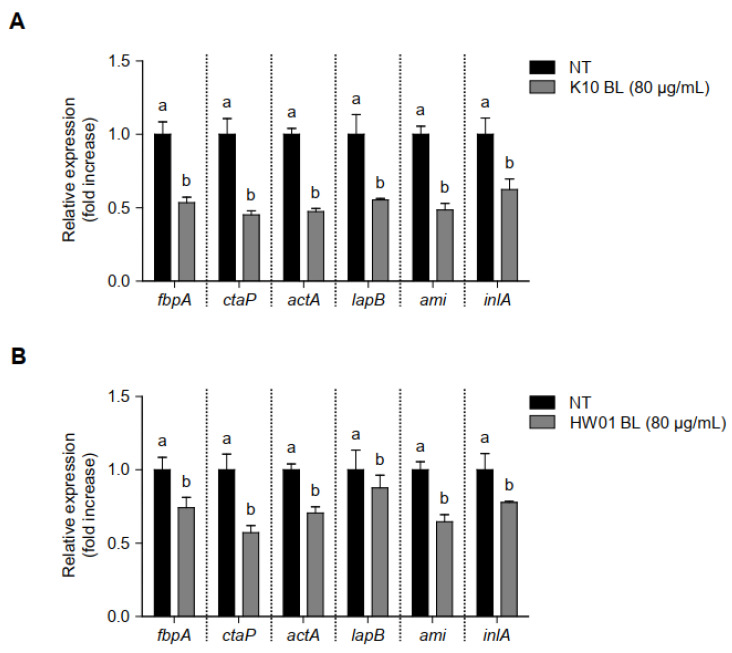
Regulation of gene transcription associated with *L. monocytogenes* adhesion and invasion in the presence or absence of *P. acidilactici* BLs. K10 BL (**A**) or HW01 BL (**B**) (80 μg/mL) was preincubated for 3 h in a culture plate, after which *L. monocytogenes* was added and cultured at 37 °C for an additional 24 h. The expression levels of *fbpA*, *ctaP*, *actA*, *lapB*, *ami*, and *inlA* were measured using qRT-PCR. The results are represented as the means ± standard deviations from three independent experiments (*p* < 0.05). Significant differences between treatment with K10 BL or HW01 BL are expressed with different letters (a and b).

To examine gene regulation responsible for adhesion and invasion in *L. monocytogenes* in the presence or absence of *P. acidilactici* BLs, the transcriptional profiles of *fbpA*, *ctaP*, *actA*, *lapB*, *ami*, and *inlA*, which are associated with adhesion and invasion of *L. monocytogenes*, were investigated. *L. monocytogenes* treated with K10 BL (80 μg/mL) significantly decreased the expression all of genes tested in this study (*p* < 0.05), compared to the untreated control (Figure 6A). Similar results were observed when *L. monocytogenes* was treated with HW01 BL (80 μg/mL); in this case, *fbpA*, *ctaP*, *actA*, *lapB*, *ami*, and *inlA* were significantly downregulated (*p* < 0.05). These results suggest that *P. acidilactici* BLs regulate the expression of genes responsible for adhesion and invasion and thus may inhibit *L. monocytogenes* adhesion to and invasion of HT-29 cells. 

## 4. Discussion

*L. monocytogenes* is a well-known invasive bacterium that causes life-threatening foodborne diseases in humans [27]. Bacterial adhesion is a crucial step in the process of biofilm formation and invasion of host cells, facilitating infection [28]. Many lactic acid bacteria, particularly *Lactobacillus* spp., have shown an ability to control *L. monocytogenes* by inhibiting its growth and biofilm formation [20,29,30]. Moreover, it has been also reported that bacteriocin-producing *P. acidilactici* inhibited the growth of *L. monocytogenes* [25,31]. However, the role of *P. acidilactici* BLs in controlling biofilm formation, adhesion, and invasion of *L. monocytogenes* has not been elucidated. In the present study, *P. acidilactici* BLs markedly inhibited *L. monocytogenes* biofilm formation as well as its adhesion to and invasion of intestinal epithelial cells. These results suggest that *P. acidilactici* BLs have the potential to act as biocontrol agents against *L. monocytogenes*. 

The ability of probiotics and their metabolites to reduce *L. monocytogenes* biofilm formation has been widely studied. For example, *Lactobacillus sakei* CRL1862 reduced the biofilm formation of *L. monocytogenes* by forming its own biofilm on abiotic surfaces [20]. Some *Lactobacillus* spp. have been reported to reduce *L. monocytogenes* biofilm development on the surfaces of lettuce and stainless steel in co-incubations of *Lactobacillus* spp. and *L. monocytogenes* [21]. In addition, bacteriocins derived from *Latilactobacillus curvatus* inhibited biofilm formation and motility of *L. monocytogenes* by regulating genes responsible for biofilm formation [22]. Moreover, the inhibitory effect of *P. acidilactici* on the growth of *L. monocytogenes* has been documented. Bacteriocins and cell-free culture supernatants of *P. acidilactici* suppressed the growth of *L. monocytogenes* [32,33,34]. However, the effect of *P. acidilactici* itself or its intracellular derivatives on the inhibition of biofilm development, adhesion, and invasion of *L. monocytogenes* has not been elucidated. Postbiotics have been recognized as favorable and promising alternatives to probiotics [18]. Postbiotics containing biological active factors frequently show health benefits for the host [35]. For instance, postbiotics derived from lactic acid bacteria exhibited the antagonistic activity against *L. monocytogenes* biofilm [36]. A similar report has also shown the antilisterial activity of cell-free supernatants from *Lactobacillus* spp. [37]. Although postbiotics derived from lactobacilli exhibited the inhibitory activity against *L. monocytogenes*, the antagonistic effect of postbiotic compounds, such as BLs, derived from pediococci has not been well documented. Here, our study demonstrated that *P. acidilactici* BLs noticeably reduced biofilm formation in *L. monocytogenes* and also inhibited its ability to adhere to and invade intestinal epithelial cells. 

Although the antibiofilm mechanism of BLs has not been established, some have speculated that probiotics’ production of substances with antagonistic properties, which could be present in the lysates of probiotics, could modify quorum sensing [38]. Moreover, during biofilm formation, several genes are involved in flagellar motility, such as *flaA*, *fliG*, and *flgE* [8]. Paenibacterin produced by *Paenibacillus thiaminolyticus* significantly reduced these biofilm-associated genes, consequently leading to the inhibition of *L. monocytogenes* biofilm formation [39]. In line with this possibility, we found that the expression of *agrA*, which is associated with virulence regulation via activation of the quorum-sensing mechanism [40], was downregulated by *P. acidilactici* BLs. This result suggests that specific substance(s) in *P. acidilactici* BLs could regulate the quorum-sensing mechanism of *L. monocytogenes*, ultimately reducing biofilm formation. Additionally, we also observed the decreased expressions of other genes, such as *flaA*, *fliG*, *flgE*, and *prfA*, involved in *L. monocytogenes* biofilm formation, suggesting that *P. acidilactici* BLs inhibits *L. monocytogenes* biofilm by affecting quorum sensing and flagellar motility. Furthermore, previous reports have shown that biofilm-inhibiting peptides contain positively charged amino acids, such as lysine [41,42]. The peptides rich in lysine form a charge clamp that enhances the proper association of biofilm-inhibiting peptides with the microbial cell membrane of target bacteria [43]. In the current study, K10 BL and HW01 BL did not effectively eradicate the planktonic *L. monocytogenes* viability. However, *P. acidilactici* BLs regulated genes responsible for biofilm formation in *L. monocytogenes*. A previous report showed that a synthetic cationic peptide did not affect the bacterial growth of *Pseudomonas aeruginosa* but effectively reduced *P. aeruginosa* biofilm formation [44]. Moreover, a recent study demonstrated that tryptophan-containing peptides modified quorum sensing and decreased biofilm development of *P. aeruginosa* [45]. These results indicate that substances such as peptides with antagonistic properties in *P. acidilactici* BLs may regulate genes involved in biofilm formation of *L. monocytogenes*. 

In addition, the current study demonstrated that *P. acidilactici* BLs can also inhibit the adhesion and invasion of *L. monocytogenes*. Secreted proteins from *Lactobacillus plantarum* BMCM12 has been reported to participate in the inhibition of pathogen adhesion to mucin layers [46]. Moreover, a soluble protein HM0539 derived from *L. rhamnosus* GG enhanced tight junction and increased mucus secretion in intestinal epithelial cells, which probably attenuated pathogen invasion by promoting intestinal integrity [47]. Thus, *P. acidilactici* BLs containing proteins or peptides could play a role in the inhibition of *L. monocytogenes* adhesion to and invasion of intestinal epithelial cells. Moreover, *L. monocytogenes* adhesion is the initial step in biofilm development, and it is critical for the establishment of infection [14]. Several proteins, including FbpA, CtaP, ActA, LapB, and Ami, are closely associated with adhesion to and invasion of host cells [14,48]. Although it has been well studied that many probiotics can inhibit *L. monocytogenes* biofilm formation on and adhesion to host cells [49,50], the role of probiotics in regulating genes responsible for the adhesion and invasion of *L. monocytogenes* has not been revealed. Our observations indicate that critical genes underlying adhesion and invasion in *L. monocytogenes* were markedly downregulated by *P. acidilactici* BLs. Among the proteins, *fbpA* gene-encoding fibronectin-binding protein is a cell-wall-anchored protein that is distributed in Gram-positive bacteria, including *L. monocytogenes,* and plays a role in the interaction between the bacterial and the host cells [51]. Adhesion of Δ*fbpA L. monocytogenes* to host cells is decreased, suggesting that fbpA is an important factor in the pathogenesis of *L. monocytogenes* [52]. Moreover, a reduction of invasion capability in Caco-2 cells was observed in Δ*inlA L. monocytogenes* [53]. These findings are in agreement with our results of the importance of fbpA and inlA in *L. monocytogenes* adhesion and invasion. In addition, although a transcription factor, prfA is involved in *L. monocytogenes* biofilm formation as described above, it is also associated with the regulation of genes, such as *inlA* and *actA* [54]. Therefore, we speculate that *P. acidilactici* BLs downregulated *prfA* genes, consequently resulting in a decrease of *inlA* and *actA* gene expression.

In conclusion, the present study showed the antagonistic activities of *P. acidilactici* BLs against *L. monocytogenes* biofilm formation. *P. acidilactici* BLs also inhibited *L. monocytogenes* adhesion to and invasion of intestinal epithelial cells. Although probiotic lactobacilli and their postbiotic compounds have widely shown the antagonistic activity against the growth and biofilm formation of *L. monocytogenes*, *P. acidilactici* BLs as postbiotic compounds also inhibit biofilm formation of *L. monocytogenes* and its adhesion to and invasion of intestinal epithelial cells. These inhibitory effects are possibly affected by downregulating genes responsible for biofilm formation as well as adhesive and invasive abilities in *L. monocytogenes*. Although further studies are needed to confirm specific peptides or proteins of *P. acidilactici* BLs that are associated with antagonistic activities against *L. monocytogenes*, these results suggest that *P. acidilactici* BLs could reduce health risks posed by *L. monocytogenes*. 

## Data Availability

The data that are presented in this study are available on request from the corresponding author.
